# Tyrosine Phosphorylation Modulates Peroxiredoxin-2 Activity in Normal and Diseased Red Cells

**DOI:** 10.3390/antiox10020206

**Published:** 2021-02-01

**Authors:** Alessandro Mattè, Enrica Federti, Elena Tibaldi, Maria Luisa Di Paolo, Giovanni Bisello, Mariarita Bertoldi, Andrea Carpentieri, Pietro Pucci, Iana Iatcencko, Anand B. Wilson, Veronica Riccardi, Angela Siciliano, Francesco Turrini, Dae Won Kim, Soo Young Choi, Anna Maria Brunati, Lucia De Franceschi

**Affiliations:** 1 Department of Medicine, University of Verona and AOUI Verona, 37134 Verona, Italy; alessandro.matte@univr.it (A.M.); enrica.federti@univr.it (E.F.); ianaiatcenko@gmail.com (I.I.); anand.wilson16@gmail.com (A.B.W.); veronica.riccardi@univr.it (V.R.); angela.siciliano@univr.it (A.S.); 2 Department of Molecular Medicine, University of Padua, 35128 Padua, Italy; elena.tibaldi@unipd.it (E.T.); marialuisa.dipaolo@unipd.it (M.L.D.P.); Annamaria.brunati@unipd.it (A.M.B.); 3 Department of Neuroscience, Biomedicine and Movement Sciences, Section of Biological Chemistry, University of Verona, 37134 Verona, Italy; giovanni.bisello@univr.it; 4 Department of Chemical Sciences, University Federico II of Napoli, 80126 Napoli, Italy; andrea.carpentieri@unina.it (A.C.); pucci@unina.it (P.P.); 5 CEINGE Biotecnologie Avanzate, 80145 Napoli, Italy; 6 Department of Oncology, University of Torino, 10124 Torino, Italy; francesco.turrini@unito.it; 7 Department of Biomedical Sciences and Institute of Bioscience and Biotechnology, Hallym University, Chuncheon 24252, Korea; dwkim@hallym.ac.kr (D.W.K.); sychoi@hallym.ac.kr (S.Y.C.)

**Keywords:** peroxiredoxin-2, tyrosine phosphorylation, Syk, sickle cell disease, oxidation

## Abstract

Peroxiredoxin-2 (Prx2) is the third most abundant cytoplasmic protein in red blood cells. Prx2 belongs to a well-known family of antioxidants, the peroxiredoxins (Prxs), that are widely expressed in mammalian cells. Prx2 is a typical, homodimeric, 2-Cys Prx that uses two cysteine residues to accomplish the task of detoxifying a vast range of organic peroxides, H_2_O_2_, and peroxynitrite. Although progress has been made on functional characterization of Prx2, much still remains to be investigated on Prx2 post-translational changes. Here, we first show that Prx2 is Tyrosine (Tyr) phosphorylated by Syk in red cells exposed to oxidation induced by diamide. We identified Tyr-193 in both recombinant Prx2 and native Prx2 from red cells as a specific target of Syk. Bioinformatic analysis suggests that phosphorylation of Tyr-193 allows Prx2 conformational change that is more favorable for its peroxidase activity. Indeed, Syk-induced Tyr phosphorylation of Prx2 enhances in vitro Prx2 activity, but also contributes to Prx2 translocation to the membrane of red cells exposed to diamide. The biologic importance of Tyr-193 phospho-Prx2 is further supported by data on red cells from a mouse model of humanized sickle cell disease (SCD). SCD is globally distributed, hereditary red cell disorder, characterized by severe red cell oxidation due to the pathologic sickle hemoglobin. SCD red cells show Tyr-phosphorylated Prx2 bound to the membrane and increased Prx2 activity when compared to healthy erythrocytes. Collectively, our data highlight the novel link between redox related signaling and Prx2 function in normal and diseased red cells.

## 1. Introduction

Peroxiredoxin-2 (Prx2) is the third most abundant cytoplasmic protein in red blood cells. Prx2 belongs to a well-known family of antioxidant proteins, the peroxiredoxins (Prxs), widely expressed in mammalian cells [1,2,3,4,5,6,7]. Prx2 is a member of the typical, homodimeric, 2-Cys Prxs, a wider group that uses two cysteine residues to accomplish the task of detoxifying a vast range of organic peroxides, H_2_O_2_, and peroxynitrite. The catalytic mechanism is characterized by a sequence of events, starting from one cysteine sulfhydryl of the first subunit that is oxidized by the substrate, to sulfenic acid (the so-called peroxidatic cysteine, Cys-S_P_H). This is then resolved by the action of a second cysteine sulfhydryl of the cognate subunit (the so-called resolving cysteine, Cys-S_R_H) through the formation of a disulfide subsequently re-reduced by the action of thioredoxin or glutathione and glutathione reductase [2,8].

In addition to the enzymatic function, Prxs have been shown to acquire a chaperone function under oxidative stress conditions, and are able to interact with multiple proteins [2,9]. Accordingly, a crucial role is played by the possibility of switching the oligomeric structure from a homodimer to a homodecamer in the reduced state, while sulfur oxidation of the two essential cysteine residues leads to decamer dissociation into disulfide-linked dimers. Hyperoxidation of the peroxidatic cysteine (Cys-S_P_O_2_H) leads to the formation of catalytically inactive, high-molecular-weight oligomers with chaperone function [2]. Since redox conditions are so crucial for cell viability, it is not strange that Prxs could be highly regulated by several modifications, such as phosphorylation, acetylation, glutathionylation, and nitrosylation. Prx1 and Prx2 exhibit particular sensitivity to post-translational changes [8,10,11].

**Figure 1:**
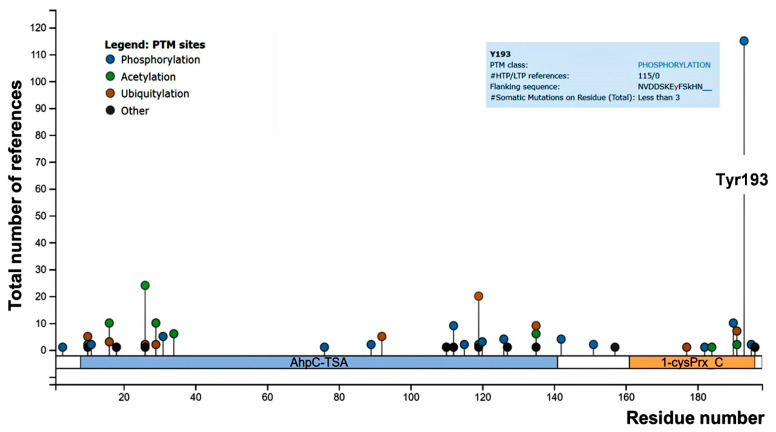
PhosphoSitePlus lollipop plot of mouse peroxiredoxin-2 (Prx2), showing the number of references for the post-translational modified sites throughout the Prx2 aminoacidic sequence. Both high (HTP) and low (LTP) throughput papers were included in the report. Tyrosine (Tyr)-193 is highlighted, and details are shown in the blue square.

Focusing on phosphorylation, Prx1 has been reported to be phosphorylated both on tyrosine (Tyr) and on threonine/serine residues. Although several phosphorylation sites of Prx1 have been identified, limited data are available on their impact on Prx1 function [10,12]. Concerning Prx2, several threonine/serine residues and only one Tyr residue (Tyr-193) have been identified as being phosphorylated [12]. We carried out a revision of the literature and found Tyr-193 to be the most frequently tyrosine residue identified as phosphorylated in Prx2, suggesting a possible biologic importance of this post-translation modification for Prx2 function (Figure 1).

Previous studies have shown that changes in the phosphorylation of Prxs might affect not only their activity, but also their molecular size, by shifting the dimer–decamer protein equilibrium to an inactive, high-molecular-weight decamer species with a chaperone function [12]. This suggests a possible link between oligomerization, peroxidase activity, chaperone activity, and post-translational modifications. 

Red cells are an exceptional model to study Prx2 function and post-translational modifications, due to their highly oxidative physiologic environment. Recently, we have shown that Prx2 is important in both red cells and erythropoiesis to ensure cell survival, growth, and differentiation against physiologic and pathologic oxidation [13,14,15,16]. In addition, we found that Prx2 might act as a chaperone-like protein targeting key membrane proteins, such as band 3, against oxidation [17,18,19]. Indeed, mice genetically lacking Prx2 display accelerated red cell senescence. This is associated with an overactivated Syk kinase-dependent intracellular signaling pathway, ending with the release of erythroid microparticles to clear damaged proteins [17,18,19,20]. In red cells, the biologic importance of redox-related signaling pathways is supported by studies in red cells from both animal models and human subjects with hemoglobinopathies or glucose 6-phosphate dehydrogenase G6PD deficiency [13,14,15,17,21,22]. In response to oxidation, Syk translocates to the membrane and favors band 3 clusterization, resulting in erythroid vesiculation followed by red cell membrane rearrangement [17,18,19]. This mechanism is important in pathologic red cells, such as in β-thalassemia, or in sickle cell disease (SCD) [14,17,20]. Both conditions are characterized by severe membrane oxidation and activation of the intracellular Syk signaling pathway [14,17,20]. In red cells from a mouse model of SCD, we previously reported a membrane translocation of Prx2 in response to oxidation related to hypoxia/reoxygenation stress, mimicking sickle cell-related vaso-occlusive crisis [22,23]. This suggests a possible connection between the redox-related signaling pathway and Prx2 in both normal red cells exposed to exogenous oxidation and pathologic erythrocytes, such as in SCD.

Here, we first found that Prx2 is Tyr-phosphorylated in response to oxidation and translocates to the membrane. We identified Tyr-193 on Prx2 as specific target of Syk kinase, resulting in increased Prx2 activity. The bioinformatic analysis revealed that Tyr-193 is involved in the conformational changes that Prx2 undergoes during catalysis. The biologic importance of phosphorylated, active Prx2 is further supported by the observation of phospho-Prx2 bound to the membrane that is associated with increased Prx2 activity in sickle red cells. Taken together, our data show that Tyr-phosphorylation of Prx2 contributes to activating Prx2, and to partitioning Prx2 between the cytoplasm and membrane, thus participating in redox-related signaling machinery linking the Syk pathway to Prx2 post-translational changes in normal and diseased red cells. 

## 2. Materials and Methods 

### 2.1. Mouse Strains and Design of the Study

The Institutional Animal Experimental Committee of University of Verona (CIRSAL) and the Italian Ministry of Health approved the experimental protocols (prot. 56DC9.21). Three-month old C57B6/2J wild-type (WT) mice and SCD (Hba^tm1(HBA)Tow^ Hbb^tm2(HBG1,HBB*)Tow^) mice were studied [24,25,26]. Whenever indicated, red cells underwent to in vitro treatments with oxidative agents or specific inhibitors [17,27,28]; details are reported in the Supplementary Materials.

### 2.2. Two-Dimensional Electrophoresis, Phosphoprotein Enrichement, Western-Blot Analysis, and Immunoprecipitation Assay

Red cell membrane (ghost) and cytosol fractions were obtained as previously reported [13,15,26,29,30] (Figure S1).

#### 2.2.1. Two-Dimensional Electrophoresis (2D) Analysis

Red cell membrane proteins underwent two-dimensional electrophoresis (2D) analysis and separated, in the first dimension, onto a 3.0–5.6 immobilized pH gradient IPG strip, and in the second dimension, by SDS-PAGE [31]. Details are in the Supplementary Materials.

#### 2.2.2. Phosphoprotein-Enriched Samples

Phosphoprotein-enriched samples were generated from red cell membrane protein extracts using a TALON PMAC Phosphoprotein Enrichment Kit (ClonTech, CA, USA) according to the manufacturer’s instructions [32,33]. 

#### 2.2.3. Western-Blot Analysis and Immunoprecipitation Assay 

Red cell membrane (ghost) and cytosol fractions were analyzed by SDS-PAGE [13,15,26,29,30]. Gels were transferred to nitrocellulose membranes for immunoblot analysis with specific antibodies. Details are in the Supplementary Materials. Immunoprecipitations of anti-phospho-Tyr proteins from red cells were carried out using Protein A Agarose (Thermo Fisher Scientific, Waltham, Massachusetts, United States), as well as a mix of the anti phospho-Tyrosine monoclonal antibodies PY99 (Santa Cruz Biotechnology, Dallas, TX, USA) and 4G10 (Merck Group, Darmstadt, Germany) as previously reported [17]. 

### 2.3. Generation of Recombinant Prx2 and In Vitro Prx2 Activity

Human WT Prx2 and mutated Prx2-Y193F and Prx2-Y115F were cloned in a pET15b vector to produce the recombinant enzymes. Details are reported in the Supplementary Materials. Prx2 activity of recombinant enzyme (both of the control and the phosphorylated enzyme) was performed according to the basic protocol IV reported in Nelson and Parsonage [34], with minor modifications (see Figure S2 and Supplementary Materials). 

### 2.4. Mass Spectrometry

Proteins were either analyzed by a Tofspec SE (Micromass, Manchester, United Kingdom) and a LTQ Orbitrap XL Hybrid Ion Trap-Orbitrap Mass Spectrometer (Thermo Fisher Scientific, Bremen, Germany). Details are reported in the Supplementary Materials. 

### 2.5. Bioinformatic Analysis

Structural analysis of the Prx2 active site in its hyperoxidized (PDB = 1qmv) and oxidized disulfide form (PDB = 5ijt) was carried out using Pymol 2.0 (PyMOL Molecular Graphics System, Version 2.0 Schrödinger Inc, New York, NY, United States). In order to simplify the representation, only a dimer for each decamer is shown. 

### 2.6. Prx2 Activity on Mouse Red Cells

Enzymatic activity of Prx2 on red cells from wild-type and humanized sickle cell [24,25] mice was assayed, as previously reported [15] in the presence of the coupled assay thioredoxin, thioredoxin reductase, and NADPH. The relative activity was determined by measuring the Δ*A*/min at 340 nm of 10 μL of samples for both WT and SCD, considering as a reference the activity of WT as 100% of peroxidase activity. 

### 2.7. Statistical Analysis

Data were analyzed using either a *t*-test or one-way ANOVA for longitudinal studies, or one-way ANOVA for multiple comparisons. A difference with *p* < 0.05 was considered significant. 

## 3. Results

### 3.1. Prx2 Is Tyrosine-Phosphorylated in Response to Oxidation and Associates to the Membrane

**Figure 2:**
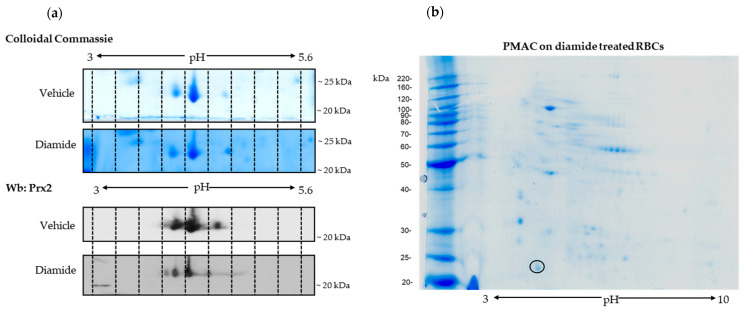
Prx2 is phosphorylated in response to oxidative stress. (**a**) Two-dimensional electrophoresis (2D) analysis of ghosts from wild-type (WT) red cells treated with vehicle or 2 mM diamide. Gradient strips of 3.0–5.6 immobilized pH were used. One representative experiment of three performed with similar results is shown. We ran twin gels, one stained with colloidal Coomassie and the other one blotted with a specific anti-Prx2 antibody. (**b**) Phospho-enriched proteins from diamide (2 mM)-treated, wild-type red cell membrane proteins were obtained by a PMAC metal affinity column and analyzed by bi-dimensional electrophoresis (2D) in a 3–10 immobilized pH gradient (right panel). Shown is a representative experiment of three performed with similar results. Gels were stained with colloidal Coomassie. The black circle indicates the spot identified as Prx2 by MALDI-TOF analysis and further confirmed by LC-MS/MS analysis. Prx2 was identified with the following peptides: RGLFIIDAKG; KNDEGIAYRG; KSLSQNYGVLKN; RQITVNDLPVGRS; K.SAPDFTATAVVDGAFKE.

To address the question of whether post-translational modification might affect Prx2 in response to oxidation, we exposed wild-type mouse erythrocytes to diamide, an oxidant known to promote red cell membrane damage, requiring Prx2 membrane translocation [13,17]. We performed 2DE analysis of the membrane fraction (ghost) from mouse red cells treated with either vehicle or diamide (2 mM) [13,14]. Prx2 was identified by mass-spectrometric analysis in both vehicle- and diamide-treated red cells (AC #Q61171, Prdx2_mouse; the vehicle matched 5 peptides, with coverage of 24%, while the Prx2 diamide matched 4 peptides, with coverage of 22%) (Figure 2a). The immunoblot analysis carried out in twin-gels revealed the presence of trains of Prx2, which were left shifted in red cells treated with diamide when compared to vehicle-treated erythrocytes, suggesting a possible post-translational Prx2 modification, such as phosphorylation (Figure 2a). This intrigued us, since previous studies on Tyr-phospho-maps of red cells from both healthy mice and human subjects did not find phospho-Tyr–Prx2 associated with the membrane [14,31]. We then carried out phospho-peptide enrichment using a phospho-specific metal ion affinity resin (TALON PMAC Phosphoprotein Enrichment Kit) of mouse red cells exposed to diamide [32,33]. Prx2 was identified by MALDI-TOF analysis and further confirmed by LC-MS/MS analysis (Figure 2b). 

**Figure 3:**
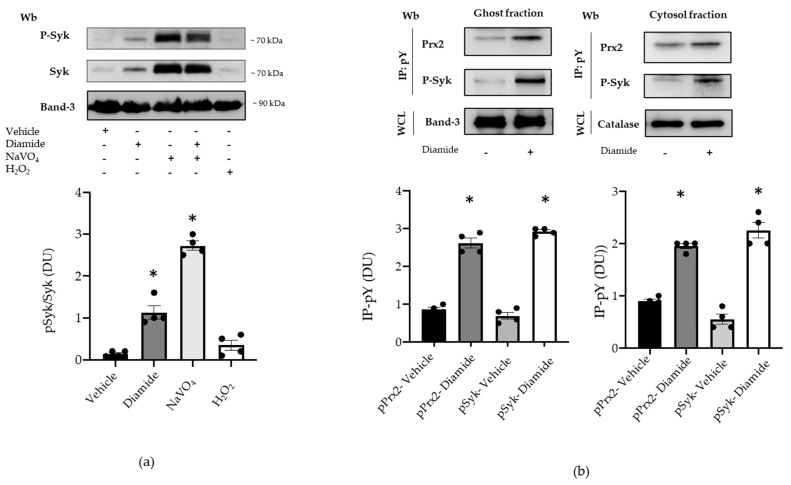
Prx2 is Tyr-phosphorylated in diamide-treated red cells, characterized by activation of the Syk canonical pathway. (**a**) Upper panel: Western blot (Wb) analysis with specific antibodies against P-Syk and Syk of the cytosolic fraction from red cells of WT mice treated with vehicle or diamide, NAVO_4_, and/or H_2_O_2_. Band 3 is used as loading control. One representative gel from other four with similar results is presented. Lower panel: densitometric analysis is presented as means ± standard error of the mean (SEM) (*n* = 4); * *p* < 0.05 compared to vehicle-treated red cells. (**b**) Upper panel: ghost and cytosol fractions from red cells of wild-type (WT) mice treated with either vehicle or diamide underwent immunoprecipitation with specific anti-phospho-tyrosine antibodies (IP: pY), and were then revealed with specific anti-Prx2 or anti-P-Syk antibodies. Band 3 and catalase, as well as colloidal Coomassie-stained gels (see Figure S3a) in whole-cell lysate (WCL), were used as loading controls. One representative gel from the other four with similar results is presented (see also Figure S3a). Lower panel: densitometric analysis is presented as means ± SEM (*n* = 4); * *p* < 0.05 compared to vehicle treated red cells.

Since Syk is the key kinase in red cell signaling networks in response to oxidation, we have explored the Syk pathway in diamide-treated erythrocyte with respect to Prx2 [17,18,19,31,35]. As shown in Figure 3a, Syk was activated in diamide-treated red cells, compared to vehicle-treated erythrocytes. The amount of active phospho-Syk was further increased in the presence of NaVO_4_, blocking families of phosphatases (Figure 3a). No major change was observed in Syk activity in H_2_O_2_-treated erythrocytes (Figure 3a) [13,17,28]. Thus, we focused on diamide-treated red cells. As shown in Figure 3b, we found increased membrane association of both Tyr-phosphorylated Prx2, corresponding to almost 47% of total membrane-bound Prx2, and Syk in response to oxidation when compared to vehicle-treated erythrocytes. This was paralleled with increased Tyr phosphorylation of Prx2, also in the cytoplasmatic compartment of diamide-treated red cells (corresponding to 3% of total cytoplasmic Prx2; Figure 3b, see also Figure S3a). 

**Figure 4:**
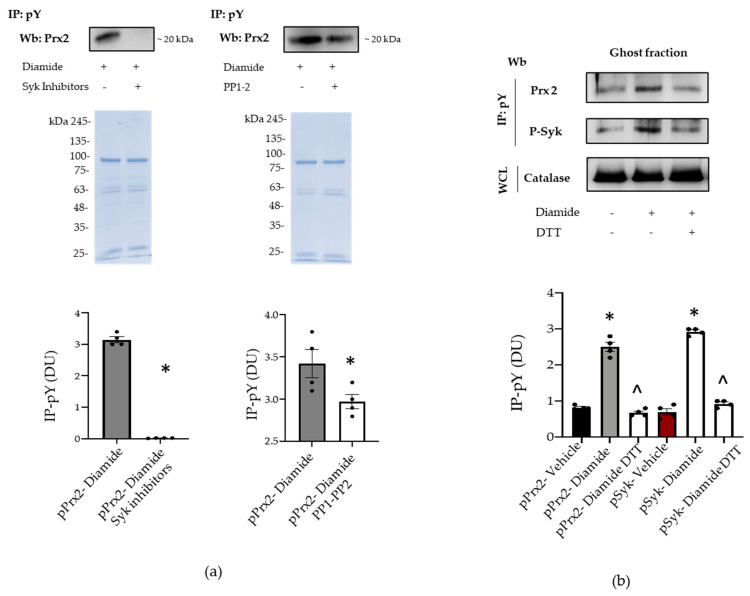
Syk inhibitors and dithiothreitol (DTT) prevents the phosphorylation of Prx2. (**a**) Ghost fraction from the red cells of wild-type (WT) mice treated with diamide and/or Syk inhibitors (I–II) and PP1–2 underwent immunoprecipitation with specific anti-phospho-tyrosine antibodies (IP: pY), and then were used for Western blot (Wb) analysis with anti-Prx2 antibodies. Colloidal Coomassie-stained gels in whole-cell lysate (WCL) were used as loading controls. One representative gel from three with similar results is presented. Densitometric analysis is presented means ± SEM (*n* = 4); * *p* < 0.05 compared to vehicle-treated red cells. (**b**) Ghost fraction from red cells of wild-type (WT) mice treated with vehicle or diamide and/or DTT underwent immunoprecipitation with specific anti-phospho-tyrosine antibodies (IP: PY), and then were used for Western blot (Wb) analysis with either anti-Prx2 or anti-P-Syk antibodies. Band 3, as well as colloidal Coomassie-stained gels (see Figure S3c) in whole-cell lysate (WCL), was used as a loading control. One representative gel from three with similar results is presented. Densitometric analysis is presented as means ± SEM (*n* = 4); * *p* < 0.05 compared to vehicle-treated red cells; ^ *p* < 0.05 compared to diamide-treated red cells.

To explore whether the membrane translocation of phospho-Prx2 was dependent on Syk activation, we used specific inhibitors for either Syk or Src family kinases, such as PP1 and PP2. As shown in Figure 4a, Syk inhibitors I–II fully blocked Prx2 phosphorylation and Prx2 membrane translocation. However, PP1 and PP2 did not modify the amount of Tyr-phosphorylated Prx2 associated with the membrane of diamide-treated red cells (Figure 4a). The oxidative dependent compartmentalization of Prx2 was further supported by the observation that dithiotretol (DTT), a known thiol group donor, prevented the membrane association of Tyr-phosphorylated Prx2 (Figure 4b) [15,21]. Noteworthily, Prx2 was associated with the membrane as both a monomer and dimer in diamide-treated red cells, and DTT again prevented Prx2 dimer formation, as previously reported by us [13,36] (Figure S3b). Taken together, our data suggest that Prx2 is Tyr-phosphorylated by Syk in response to oxidation, binding to the membrane of diamide-treated red cells. 

### 3.2. Syk Phosphorylates Prx2, Resulting in an Increase in Prx2 Activity 

**Figure 5:**
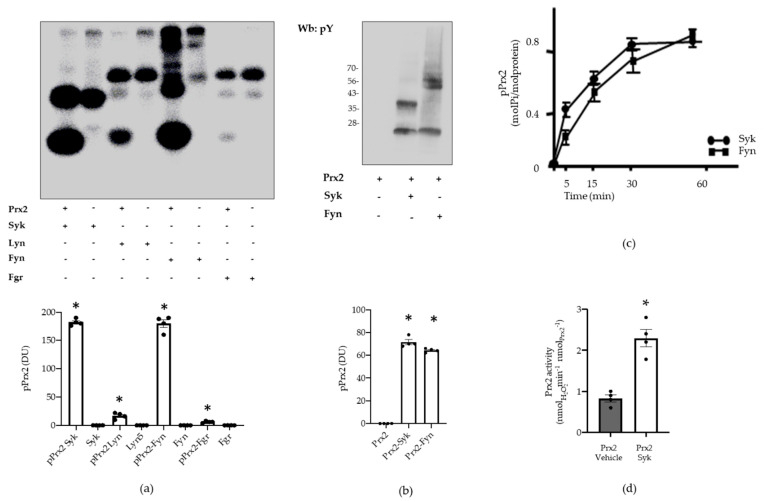
Prx2 activity is affected by Syk-mediated phosphorylation. (**a**) Upper panel: Prx2 (0.4 μg) was phosphorylated in the presence or absence of either Syk (lanes 1–2), Lyn (lanes 3–4), Fyn (lanes 5–6), or Fgr (lanes 7–8) (see Materials and Methods for further details) for 10 min, resolved by SDS-PAGE, and visualized by autoradiography. Lower panel: densitometric analysis is presented as means ± SEM (*n* = 3); * *p* < 0.05 compared to vehicle-treated red cells. (**b**) Upper panel: tyrosine phosphorylation of Prx2 and autophosphorylation of Syk or Fyn was assessed by Western blot analysis with anti-phosphotyrosine antibody after separation on SDS-PAGE, transfer onto nitrocellulose, and reveal by ECL peroxidase assay. Lower panel: densitometric analysis is presented as means ± SEM (*n* = 3); * *p* < 0.05 compared to vehicle-treated red cells. (**c**) Time course of Prx2 phosphorylation by Syk and Fyn. Values are the mean ± SEM of four determinations performed at each time point. (**d**) Enzymatic activity of Prx2 before (vehicle) and after phosphorylation by Syk. Values are the mean ± SEM (*n* = 4; * p < 0.01 compared to control Prx2 by Student’s *t*-test).

To define the contribution of Syk in the phosphorylation of Prx2, recombinant Prx2 underwent in vitro phosphorylation assays in the presence of equal units of Syk or some of members of the Src family (Src family kinases, or SFKs), which are known to be sensitive to PP1/PP2s, such as Lyn, Fyn, or Fgr [37,38] (see Materials and Methods for experimental details and the source of the enzymes). The mixture was then resolved by SDS-PAGE and visualized by autoradiography. As shown in Figure 5a, Syk was able to abundantly phosphorylate Prx2 (lane 1), whereas out of the SFKs added to the kinase mixture, Fyn only phosphorylated Prx2 to a comparable extent (lane 5). On the other hand, the Prx2 phosphorylation by Lyn and Fgr was negligible (lanes 3 and 5). These results support the notion that the substrate specificity of SFKs can widely vary, as further highlighted recently [36,37,38]. That the incorporation of ^32^P labels had occurred on tyrosine residues was further confirmed by Western blot analysis of Prx2 with anti-phospho-tyrosine antibodies (Figure 5b). Afterwards, to determine the level of incorporation of phosphate onto Prx2, time-course phosphorylation in the presence of either enzyme was performed for one hour, with single aliquots being drawn at discrete time points to be analyzed by autoradiography after SDS-PAGE (data not shown). The bands corresponding to Prx2 were excised, and the ^32^P radioactivity was determined by Cerenkov counting (Figure 5c, upper panel), the molar ratio of phosphate bound to Prx2 being found to be approximately 0.88:1.00. In addition, to assess the role of phosphorylation on Prx2 by Syk, the peroxidase activity was determined on Prx2 previously subjected to nearly exhaustive phosphorylation under the conditions described above. Results shown in Figure 5c clearly highlight that the phosphorylation of Prx2 increases its enzyme activity 2.3-fold, compared to the non-phosphorylated form of the enzyme. Noteworthily, the Prx2-specific activity here reported is comparable to that described for other Prx preparations with the same assay method, using DTT as a reducing agent [39]. Whereas we used the Trx–TrxR reducing system in red cell preparation (see Figure below). This method usually obtains more highly specific activities compared to the DTT system [39]. Independent of the method, the main result to consider is the increase of Prx2 activity, due to the phosphorylation of the enzyme compared to the non-phosphorylated form. 

### 3.3. Syk Specifically Targets Tyr-193 Residues on Prx2

**Figure 6:**
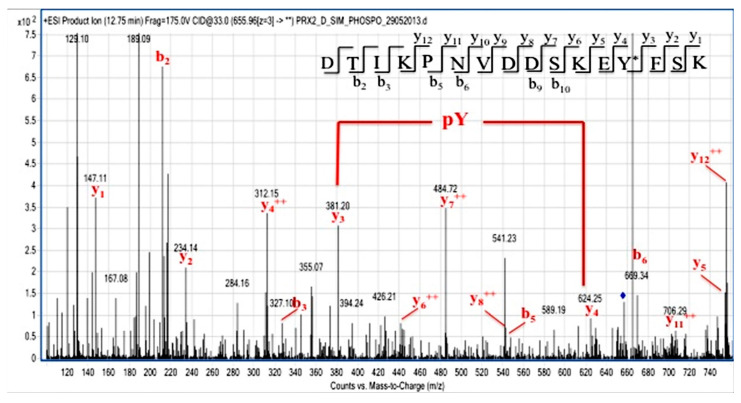
Identification of Tyr-193 on Syk-phosphorylated recombinant Prx2. Partial MS/MS spectrum of the triply charged ion at *m*/*z* 655.96 from the tryptic and AspN double digest of Prx2 following Syk incubation. Manual inspection of the y and b fragment ion series confirmed the peptide sequence. The mass difference between the y_4_ and y_3_ ions unambiguously indicate the occurrence of a phosphoTyr residue at position 193.

We then carried out phospho-proteomic analysis of recombinant Prx2 in vitro, phosphorylated by either Syk or Fyn, a Tyr-kinase of the Src family. Following Syk incubation, Prx2 was digested with trypsin and AspN, and the resulting peptide mixture was directly analyzed by LC-MS/MS. Mass spectral data showed the occurrence of a triply charged mass signal at *m*/*z* 655.96, corresponding to the peptide DTIKPNVDDSKEYFSK, with a mass increase of 80 Da, thus suggesting the presence of a phosphate group. Figure 6 shows the partial MS/MS spectrum of the triply charged ion at *m*/*z* 655.96, showing the occurrence of both the y and b ion series that confirmed the peptide sequence. As highlighted in the figure, the 243 Da mass difference between the y ions at *m*/*z* 624.25 (y_4_) and 381.20 (y_3_) unambiguously located the phospho-Tyr residue at position 193. Noteworthily, Tyr-193 was also identified in immune-enriched Prx2 from diamide-treated red cells, supporting the biologic importance of Tyr 193 in Prx2 function against oxidation. 

Similarly, the LC-MS/MS analysis of the tryptic peptides from Prx2 incubated with Fyn revealed the occurrence of a mass signal at *m*/*z* 630.31 that was assigned to the doubly charged phosphorylated peptide RLSEDYGVLK bearing a phosphate group. The corresponding MS/MS spectrum reported in Figure S4 showed the occurrence of a phosphoTyr residue at position 115, as demonstrated by the mass difference of 243 Da between the y ions at *m*/*z* 844.32 and 601.30.

### 3.4. Tyr-193 Is Positioned in Proximity to the Active Site of Prx2

**Figure 7:**
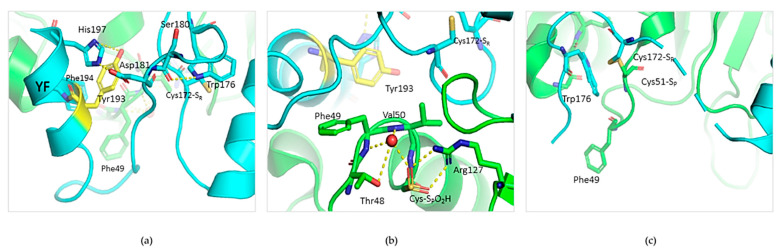
Structural environment of Tyr-193 in reduced and oxidized Prx2. (**a**) Structural features of the active site of reduced Prx2 evidencing one monomer (in cyan) and highlighting (in yellow) Tyr-193 in the hydrogen bond network stabilized with the resolving Cys-172. (**b**) Insight into the network existing from Tyr-193 to the peroxidatic Cys51, here in its sulfinic form (from PDB 1qmv). The green color refers to the neighboring subunit. (**c**) Active site rearrangement in the oxidized form of Prx2 (from PDB: 5ijt).

A structural inspection of Prx2 in its hyperoxidized (mimicking the reduced) state shows that Tyr-193 is part of an extensive hydrogen bond network in place concurrent with both the resolving Cys-172 (Figure 7a) and the buried catalytic Cys-51 (present in this structure as hyperoxidated C-S_P_O_2_H)) (Figure 7b). In detail, Tyr-193 belongs to the important structural YF motif on the C-terminal helix interacting with Phe-194, and is base-stacked to His-197 and on the same helix. His-197, in turn, stabilizes a loop containing Trp-176 by subsequential interactions with Asp-181 and Ser-180. In this conformation, Cys-51 and Cys-172 are ~13.4 Å distant, as visible in Figure 7b. Sulfinic Cys-51-S_P_-O_2_H (mimicking Cys-S_P_H-51) is buried into the active site and stabilized by a network of interactions with Arg-127, and with a water molecule that is coordinated also by Thr-48, Phe-49, and Val-50. The formation of the inter-subunit disulfide bond (Cys-51–S_P_–S_R_–Cys-172) leads to a local unfolding of the C-terminal region: the Trp-176 of one monomer moves ~18 Å apart and is packed in proximity of the GGLG motif of the second monomer, as shown in Figure 7c. The rearrangement of the C-terminal coil causes a disruption of the Tyr-193 interactions, and the YF motif completely loses its helical structure and electron density. Arg-127 is displaced, and Phe-49 is shifted 5.4 Å. 

Based on our experimental data and the bioinformatic analysis, we argue that negative charges introduced on the hydroxyl moiety of Tyr-193 upon phosphorylation would on one side preserve the polar environment, and on the other impair the extensive network of interactions of the C-terminal domain. It follows that phosphorylated Try-193 could positively concur with the fully folded-to-locally unfolded conversion.

### 3.5. Red Cells from a Humanized Mouse Model of Sickle Cell Disease Display Tyr-Phosphorylated Prx2 Associated with the Membrane 

To understand the biologic importance of Prx2 Tyr-phosphorylation in pathologic red cells, we studied erythrocytes from a humanized mouse model of SCD [24,25]. 

**Figure 8:**
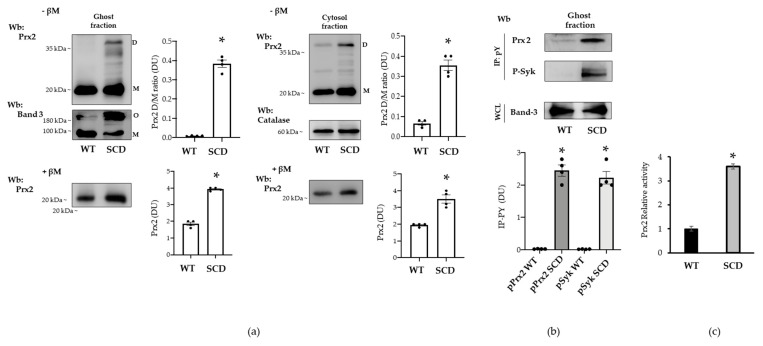
Prx2 is Tyr-phosphorylated in red cells from humanized mouse model for sickle cell disease. (**a**) Western blot (Wb) analysis with specific antibodies against Prx2 of ghost and cytosolic fractions from red cells of WT and SCD mice with or without β-mercaptoethanol (βM). Band 3 and catalase were used as loading controls. M: monomers, D: dimers, O: oligomers. One representative gel from three with similar results is presented. Densitometric analysis is presented as means ± SEM (*n* = 4); * *p* < 0.05 compared to vehicle-treated red cells. (**b**) Ghost fraction from red cells of WT and SCD mice underwent immunoprecipitation with specific anti-phospho-tyrosine antibodies (IP: PY), and then were used for Western blot (Wb) analysis with anti-Prx2 and P-Syk antibodies. Band 3, as well as colloidal Coomassie-stained gel (see Figure S5b) in whole-cell lysate (WCL) were used as loading controls. One representative gel from three with similar results is presented. Densitometric analysis is presented as means ± SEM (*n* = 4); * *p* < 0.05 compared to vehicle-treated red cells. (**c**) Prx2 activity of WT and SCD mouse cell ghosts measured as described under Materials and Methods section. Activity of WT was considered as 100%. Results were repeated three times with a standard error less than 5%.

SCD is a globally distributed hereditary red cell disorder. SCD is characterized by presence of the pathologic hemoglobin S (HbS), which polymerizes when deoxygenated [24,25,40,41,42]. This promotes a severe red cell membrane oxidative damage, resulting in activation of Syk, aggregation of band 3, and membrane translocation of Prx2 [14,22]. In red cells from humanized SCD mice, we observed increased membrane translocation of Prx2 when compared to healthy mice (Figure 8a). Prx2 was present as both a monomer and dimer only in sickle red cells when compared to healthy erythrocytes. Noteworthily, we observed high molecular polymers of band 3 again in SCD red cells when compared to healthy erythrocytes, in agreement with previous reports on membrane oxidation of SCD red cells [22] (Figure 8a). Indeed, we found sulfonate Prx in both the cytosol and membrane compartments in SCD red cells, but not in healthy erythrocytes (Figure S5a). We then evaluated the amount of phospho-Prx2 in both healthy and SCD erythrocytes. As shown in Figure 8b, we observed membrane translocation of both active Syk and Tyr-phosphorylated Prx2 only in SCD red cells. The enzymatic activity of Prx2 was increased by a factor of 3.6-fold in SCD red cells with respect to healthy erythrocytes, underlining the contribution of overactivation of Prx2 as phosphoPrx2 to face severe oxidation in SCD red cells. 

## 4. Discussion

Here, we show for the first time that Prx2 is Tyr-phosphorylated in red cells in response to either exogenous oxidation by diamide or endogenous oxidation, such as in sickle red cells. We found that Syk specifically phosphorylates Prx2 at Tyr-193, resulting in increased Prx2 activity. This is extremely interesting, since Prx2 is the third most abundant protein in red cells, playing a key role in the antioxidant system and as an atypical chaperone in normal and diseased erythrocytes [17,36,40]. 

To date, Prx2 function has been mainly related to the redox state of the essential cysteine residues that are directly involved in the peroxidase activity of Prx2 in response to oxidation. Our in vitro studies with recombinant Prx2 have allowed us to identify Syk and Fyn as candidate kinases capable of phosphorylating Prx2. Indeed, we found Syk to phosphorylate Tyr-193 and Fyn to phosphorylate Tyr-115 residues on Prx2. Our attention was captured by Syk, since the amount of Prx2 phosphorylated by this Tyr kinase in vitro was larger than that phosphorylated by Fyn (Figure 5). In addition, Tyr-115 seems less important in relation to Prx2 function, since this residue is located in a hydrophobic cleft remote from the active site, its position remaining unchanged both in the reduced and oxidized form of Prx2 (Figure S6). On the other hand, Tyr-193 is situated in a key region, both for the catalytic activity and for the control of the oligomeric structure. Indeed, Syk-mediated Tyr-phosphorylation of Prx2 enhances Prx2 activity, as observed using recombinant Prx2 and confirmed by testing the activity of Prx2 from red cells exposed to oxidation (diamide). Tyr-193 is highly mobile, shifting from the ordered, fully folded, reduced state to the locally unfolded, disulfide-oxidized form, as shown by the bioinformatic analyses; it is able to re-order at the end of the catalytic cycle. If the sulfenic cysteine undergoes hyper-oxidation, Tyr-193 is responsible for an extensive network of H bonds, which help to stabilize the decameric assembly, thus the chaperone activity. It is possible that Tyr-193, when phosphorylated, contributes less to the rigid, fully folded form, thus allowing catalysis to occur in a favorite manner. In this sense, its post-translational, modified state helps to keep red cells more protected in highly oxidative conditions, preventing hyper-oxidation by increasing the partition ratio towards peroxidase rather than chaperone activity. In this sense, the oligomer-to-dimer equilibrium is more easily achieved at each catalytic cycle, and high molecular weight aggregates are unfavorably generated. 

The biologic importance of Tyr-193, and of its neighboring residues Lys-196 or Phe-194, located in the C-terminus of Prx2, has been also suggested by studies, both in the recombinant form and in different cell lines. Although functional studies are limited, Randall et al. have reported that Prx2 nitration on Tyr-193 results in increased Prx2 activity, associated with increased resistance of Prx2 to overoxidation [12,43]. In our model, the effects of the phosphorylation of Tyr-193 on Prx2 activity are presented using both native and recombinant Prx2. Interestingly, there is an increase in activity following the phosphorylation of Tyr-193, analogous to nitration of the same residue [43], thus suggesting that the modification of this residue, due to the addition of a negative moiety and the related structural effect, could play a key role in strengthening the peroxidase activity.

Since red cells should survive 120 days in the peripheral circulation, an efficient antioxidant and chaperone machinery is required. The functional link between Syk and Prx2 ends in empowering Prx2 activity and contributes to ensure the survival of red cells against exogenous or endogenous oxidation, respectively induced by oxidative agents, such as diamide, or pathologic hemoglobin, such as in SCD. 

**Figure 9:**
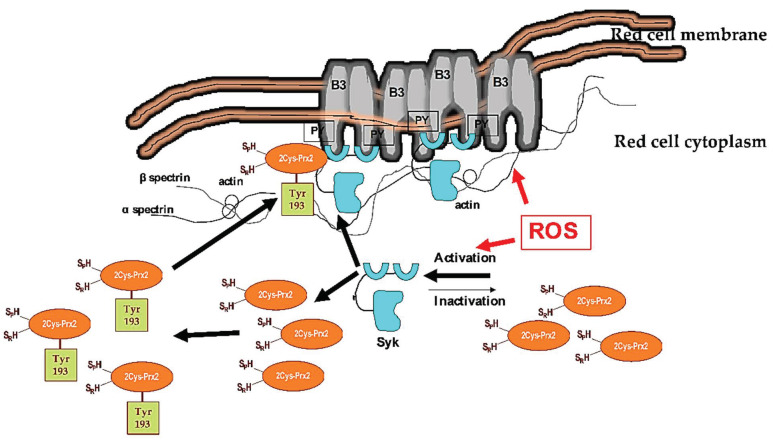
Schematic diagram of the functional model linking Syk activation in response to oxidation and Prx2 in red cells. Under steady-state conditions, Syk is inactive. Cellular stress, such as exogenous oxidation (ROS: reactive oxygen species) triggers Syk activation, resulting in increased tyrosine (Tyr) band 3 (B3) phosphorylation (PY). This modulates B3 interaction with neighboring proteins, such as band 4.1, band 4.2, adducin, or ankyrin. These multiprotein complexes bridging the membrane to the cytoskeleton contribute to the membrane’s mechanical instability. Diseased red cells, such as in sickle cell disease (SCD), are characterized by severe oxidation. This results in membrane damage and the overactivation of Syk. The damaged, over-Tyr-phosphorylated B3 forms clusters, which favor erythroid vesicle release and the fast removal of the damaged red cells by splenic macrophages. Under oxidative stress, Syk targets also Prx2. This affects both the compartmentalization of Prx2 to the red cell membrane and Prx2 activity, representing a back-up mechanism to locally minimize oxidative damage of the membrane proteins, such as band 3.

In erythrocytes, Syk plays a crucial role in transduction cell signaling against oxidation. We have previously shown that Syk overactivation favors band 3 clusterization, allowing the release of erythroid microparticles and red cell membrane rearrangement. In addition, oxidation might also affect band 3 function as an anion exchanger [27,44,45]. Exogenous antioxidants, such as curcumin or melatonin, as well as the potentiation of endogenous cytoprotectors like PEP-Prx2, might prevent or limit band 3 oxidative damage, preserving band 3 structure and function [17,44,45]. Here, we observe that diamide strongly activates Syk, whereas H_2_O_2_ slightly changes the amount of active Syk. Noteworthily, diamide targets proteins thiol groups promoting S-thiolation, whereas H_2_O_2_ irreversibly oxidizes sulfhydryl groups to sulfinic or sulfonic acid, leading to a peroxidase-incompetent Prx2. Indeed, diamide promotes diamide cross-linking of band 3 with generation of oligomeric structures that are Tyr-phophorylated by Syk [18,19]. This requires that phospho-Prx2, the more active form of Prx2, is located at the red cell membrane. Indeed, our observations suggest that Tyr-phosphorylated Prx2 translocates to the membrane. However, we cannot exclude Tyr phosphorylation of the small amount of Prx2 already bound to the membrane, as the method employed is not sensitive enough to dissect these events. The membrane localization of more active Prx2 might represent a back-up mechanism to locally minimize oxidative damage of membrane proteins, such as band 3. In agreement, band 3 was found mainly in oligomeric state, as clusterized band 3, in sickle red cell membrane, which showed increased phospho-Prx2 bound to the membrane (Figure 9). 

Our data are also important in the unique biologic context of malaria, when *Plasmodium* imposes a strong oxidative stress on the host red cells [19,35,46]. To counteract this burden, the infected erythrocytes over-activate Syk that promotes band 3 clusterization with the release of erythroid microparticles. The erythrocyte ability to face oxidation is also vital for parasite blood stage. Noteworthy, *Plasmodium* has been reported to recruit Prx2 from red cells, most likely to potentiate its own defense [47,48]. The importance of Prx2 for *Plasmodium* is further supported by the observation that parasite growth is prevented in red cells with inhibited Prx2 [47,48]. Recently, we reported that pharmacologic Syk inhibitors, such as imatinib, might represent a novel and interesting new therapeutic option for the design of host-directed intervention strategies. Imatinib potentiates the action of standard malaria treatment with artemisinin, allowing more rapid parasite clearance [19,35,46]. Our novel data on Syk-dependent Tyr phosphorylation of Prx2 add a new piece of knowledge to the molecular mechanisms of malaria infestation, emphasizing at least two functional targets of therapeutic Syk inhibition in malaria parasite red cells, namely band 3 and Prx2. 

Due to the ever-increasing knowledge regarding this complex family of biological sensors, the Prxs, endowed with both catalytical and regulation activities, this study might offer new important information on Prx2 function and regulation, as well as its crosstalk with red cell membranes in normal and diseased red cells, such as with SCD. However, some other points in the progression of this investigation could be addressed, such as the absence of mutagenesis experiments and the development of an anti-phspho-Tyr-193 Prx2 antibody to be used in different experimental conditions, in order to further functionally characterize the role of Tyr-193 Prx2 in different pathologic red cells. In addition, it is possible that phosphorylated Prx2 is a small fraction of the total pool, and the site of phosphorylation (cytosol or membrane) of Prx2 has not been identified. These points are worth addressing in future investigations.

In fact, further studies are required to fully characterize phospho-Prx2 in both normal and diseased red cells. In addition, the potentiation of endogenous antioxidant systems by the modulation of intracellular signaling might represent an interesting new frontier of investigation. 

## Data Availability

Data is contained within the article or Supplementary Material.

## Supplementary Materials

The following are available online at https://www.mdpi.com/2076-3921/10/2/206/s1, Figure S1. Colloidal Coomassie stained gels of ghost and cytosol fraction from red cells of wild-type (WT) mice treated with vehicle or diamide, Figure S2. Prx2 activity on hydrogen peroxide concentration. Dependence of the rate of reaction of the recombinant Prx2 (5 μM) at various hydrogen peroxide concentration, using DTT (200 μM) as reducing agent, Figure S3. (a) Western-blot (Wb) analysis with specific antibody against Prx2 of supernatant from immuno-precipitation assays, Figure S4. Identification of Tyr115 on Fyn phosphorylated recombinant Prx2, Figure S5. Western-blot (Wb) analysis with specific antibodies against Prx SO3, Figure S6. Localization of Tyr115 in the hyperoxidized (mimicking the reduced state) and oxidized Prx2, Methods.
