# Supplementary material for: Tyrosine Phosphorylation Modulates Peroxiredoxin-2 Activity in Normal and Diseased Red Cells
Source: Antioxidants (Basel). 2021 Feb 1;10(2):206. doi: 10.3390/antiox10020206 (PMC7912311; doi:10.3390/antiox10020206)
Supplement: Supplementary file 1 [file antioxidants-10-00206-s001.pdf]

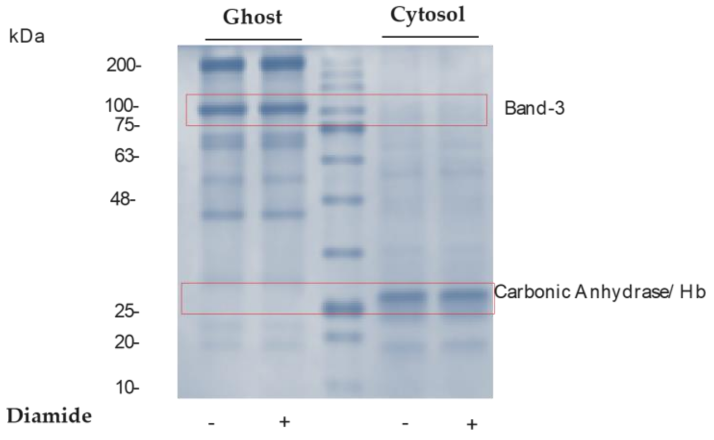

Figure S1. Colloidal Coomassie stained gels of ghost and cytosol fraction from red cells of wild-type (WT) mice treated with vehicle or diamide showing the purity of either membrane compartment (band 3- membrane integral protein) or cytoplasm compartment (carbonic anhydrase as cytoplasmic protein). One representative gel of other 4 with similar results (see also Dodge JT, Arch Biochem Biophys 100: 119, 1963).

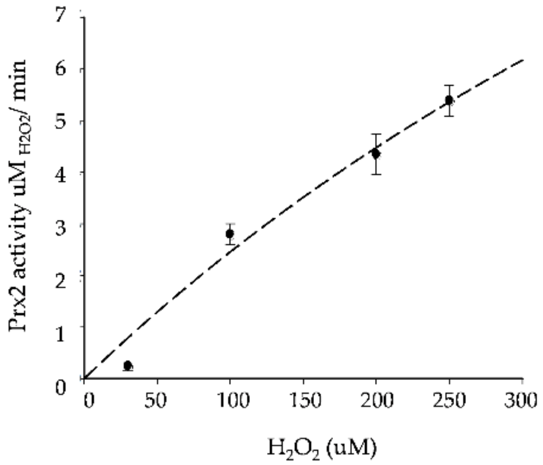

Figure S2. Prx2 activity on hydrogen peroxide concentration. Dependence of the rate of reaction of the recombinant Prx2 (5 μM) at various hydrogen peroxide concentration, using DTT (200 μM) as reducing agent.

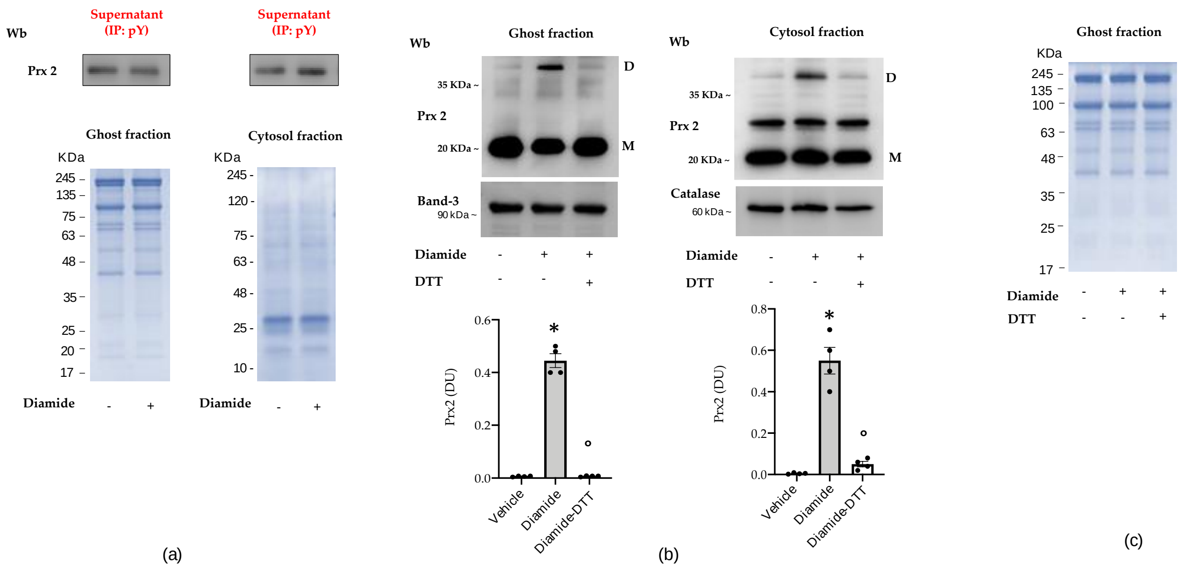

Figure S3. (a) Upper panel. Western-blot (Wb) analysis with specific antibody against Prx2 of supernatant from immunoprecipitation assays (IP) using anti-phospho-Tyrosine antibody of ghost and cytosolic fractions from red cells of wild-type (WT) mice treated with vehicle or diamide as reported in Figure 3b. **Lower panel.** Colloidal Coomassie stained gels of ghost and cytosol fractions from red cells of wild-type (WT) mice treated with vehicle or diamide used as loading controls of the immunoprecipitation reported in Figure 3b. Each one is representative of 3 separate experiments with similar results. **(b)** Western-blot (Wb) analysis with specific antibody against Prx2 of ghost and cytosolic fraction from red cells of WT mice treated with vehicle or diamide and/or DTT. Band-3 and catalase are used as loading controls. The experiment shown is representative of 3 experiments with similar results. **(c)** Colloidal Coomassie stained gels in ghost fraction from red cells of WT mice treated with vehicle or diamide and/or DTT used as loading controls of the immunoprecipitation reported in Figure 4b. Each one is representative of 3 separate experiments with similar results.

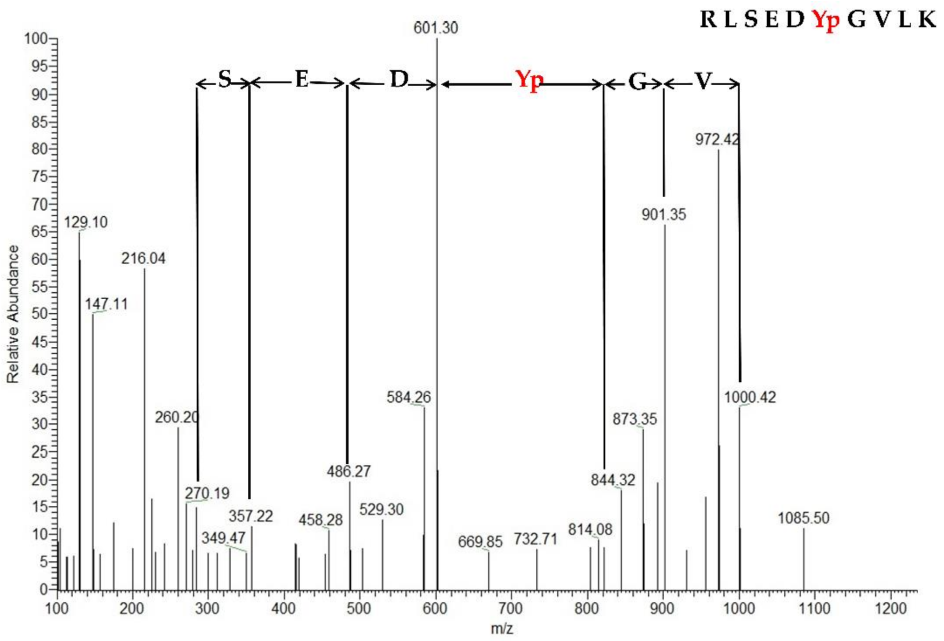

Figure S4. Identification of Tyr115 on Fyn phosphorylated recombinant Prx2. Partial MS/MS spectrum of the doubly charged ion at m/z 630.31 from the tryptic digest of Prx2 following Fyn incubation. The MS/MS spectrum showed the entire series of b ions that confirmed the peptide sequence. The occurrence of a phosphorylated Tyr residue at position 115 was indicated by the b ions at 844.32 and 601.30.

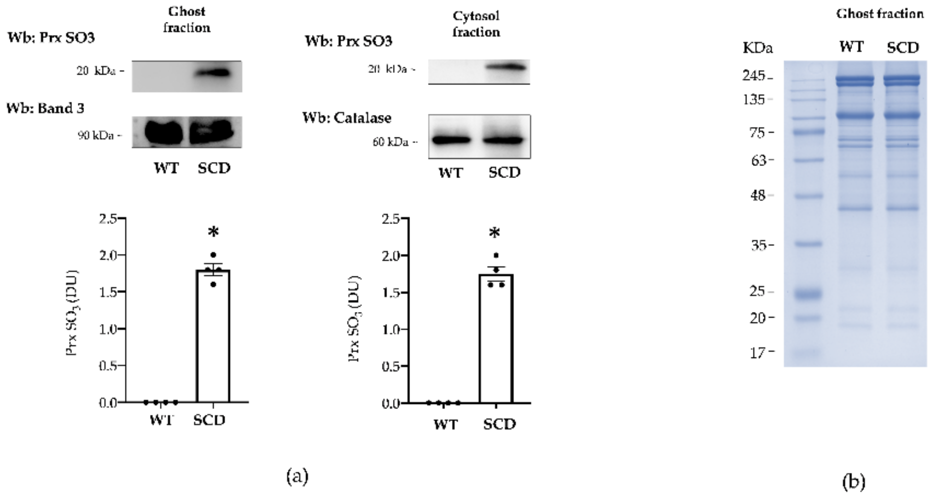

Figure S5. (a) Western-blot (Wb) analysis with specific antibodies against Prx SO_3 of ghost and cytosol fraction from red cells of WT and SCD mice. Band-3 and catalase were used as loading controls. One representative gel from other 3 with similar results is presented. **(b)** Colloidal Coomassie stained gels in ghost fraction from red cells of WT and SCD mice used as loading controls of the immunoprecipitation reported in Figure 8b. The experiment reported is representative of 3 separate experiments with similar results.

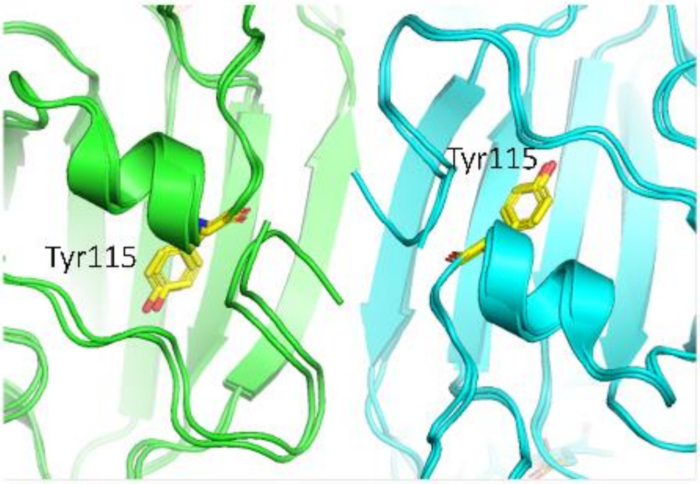

Figure S6. Localization of Tyr115 in the hyperoxidized (mimicking the reduced state) and oxidized Prx2. Tyr115 (yellow) is located in a hydrophobic cleft far from the active site and appears to maintain a similar positioning in the two superimposed structures (pdb 1qmv and 5ijt). One monomer is coloured cyan and the other monomer is green.

Methods

Mouse Strains and Design of the Study

The Institutional Animal Experimental Committee of University of Verona (CIRSAL) and the Italian Ministry of Health approved the experimental protocols (prot. 56DC9.12). Three-months old C57B6/2J wild-type (WT) mice and SCD ($Hba^{tm1(HBA)Tow}$ $Hbb^{tm2(HBG1,HBB^{*})Tow}$) mice were studied. For in vitro treatments, mouse red cells were treated with vehicle or 2 mM diamide or 75 μM H_2O_2 at 37 °C for 20 min in plasma like solution (155 mM NaCl, 3 mM KCl, 1 mM $MgCl_2$, 10 mM Tris-MOPS pH 7.4 at 37 °C, 10 mM Glucose, 1 mM K2HPO4 pH 7.4, 0.4 mM Inosine, 0.6 mM Adenine; 320 mOsm) as previously reported [1,2]. Whenever indicated red cells were pre-incubated at 37 °C for 20 min in preservation buffer (155 mM KCl, 1 mM NaCl, 0.25 mM KPO_4 pH 7.4, glucose 1 mM; 320 mOsm) at 3% Hct with

0.1 mM $NaVO_4$ or 10 mM DTT. 0.1 mM $NaVO_4$ or 10 mM DTT were then added in plasma like solution with the oxidative agents. Where indicated mouse red cells were *in vitro* pre-treated in the preservation buffer for 1 h with 10 μM Syk inhibitor II and 10 μM Syk inhibitor IV [3] or for 20 min with the Src family kinase inhibitors PP1 and PP2 at the dosage of 10 μM [4].

Two-Dimensional Electrophoresis (2D) Analysis.

600 μg of red cell membrane proteins were delipidized using a 1:12:1 ice-cold mixture of tri-n-butylphosphate, acetone and methanol, respectively. Proteins were solubilized in 2D sample buffer (2D-SB: 7 M urea, 2 M thiourea, 2% CHAPS, 2% *v/v* Triton, 40 mM Tris-HCl pH 8.8, 1% *w/v* DTT, 2.6% *v/v* ampholite carrier solution, 0.25% *w/v* Na-deoxycholate) and separated in the first direction according to isoelectric point (Ip) in a 3–10 or 3–5.6 immobilized pH gradient IPG strip (Merck Group, DE). Separation in the second direction was then performed based on molecular weight (Mw) by SDS-PAGE. Gels were either stained with Colloidal Coomassie or transferred to nitrocellulose membranes for immuno-blot analysis with a specific anti Prx2. 2D maps were analyzed with Progenesis Samespot software (NonLinear Dynamics, UK) [4].

Phosphoprotein Enriched Samples.

Phosphoprotein-enriched samples were generated from red cell membrane protein extracts using TALON PMAC Phosphoprotein Enrichment Kit (ClonTech, CA, USA) according to the manufacturer's instructions [5,6]. Briefly, 7 mg red cell membrane protein obtained by pooling blood from 4 mice were precipitated with 7.5% (*v/v*) tricloroacetic acid (TCA), incubated over-night at 0 °C and then centrifugated at 13,000× *g* at 4 °C. Pellets were washed once with 5% (*v/v*) TCA and 4 times with 80% (*v/v*) acetone, after which the proteins were dried and solubilized for 2D analysis.

Western-Blot Analysis.

Packed red cells were lysed in ice-cold phosphate lysis buffer (5 mM Na_2HPO_4, pH 8.0, containing protease inhibitor cocktail tablets, 3 mM benzamidine final concentration, 1 mM Na_3VO_4 final concentration) and centrifuged 10 min at 4 °C at 12,000× *g*. Red cell membrane (ghost) and cytosol fractions were obtained as previously reported [7–10] Proteins were quantified and analyzed by one-dimensional SDS–polyacrylamide gel electrophoresis. Gels were transferred to nitrocellulose membranes for immunoblot analysis with specific antibodies: anti Prx2 (kindly gift of Prof. Chae HZ, Chonnam National University, South Korea); anti phospho-Syk (Tyr525/526), anti Syk from Cell Signaling Technology (Danvers, USA); anti Band3 IVF12 (from Developmental Studies Hybridoma Bank, DSHB, University of Iowa, USA); anti catalase and anti Prx SO_3 from AbCam (Cambridge, UK). Secondary donkey anti-rabbit IgG and anti-mouse IgG HRP conjugated were from GE Healthcare Life Sciences (Little Chalfont, UK). Blots were developed with Luminata Forte Chemiluminescent HRP Substrate from Merk Millipore (Burlington, MA, USA), and images were acquired with the Alliance Q9 Advanced imaging system (Uvitec, UK). Densitometric analyses were performed with the Nine Alliance software (Uvitec, UK).

Generation of Recombinant Prx2 and In Vitro Prx2 Activity.

The cDNA sequence for human Prx2 was PCR-amplified using the sense primer 5′-CTCGAGATGGCCTCCGGTAA-3′, contains an XhoI site, and the antisense primer 5′-GGATCCCTAATTGTGTTTGGAGAAATA-3′, contains a BamHI restriction site. The resulting PCR products were subcloned in a TA cloning vector and ligated into a pET15b vector to produce WT Prx2. We have applied PCR based site-directed mutagenesis to change the codon of Tyr-193 of F. Prx2-Y193F was amplified by PCR using the WT Prx2 template, sense primer 5′-CTCGAGATGGCCTCCGGTAA-3′ and the antisense primer 5′-GGATCCCTAATTGTGTTTGGAGAAAAATTC-3′. The subsequent process is the same as the WT Prx2. We commissioned Bioneer (Korea Daejeon) to manufacture Prx2-Y115,193F mutant type and cloned the pET15b vector. Prx2-Y115F was amplified by PCR based site-

directed mutagenesis using the Prx2-Y115,193F template and primer WT Prx2. The completed PCR product has a Tyr-115 of F. The subsequent process is the same as the WT. Prx2 activity of recombinant enzyme (both of the control and the phosphorylated enzyme) was performed by measuring the disappearance of hydrogen peroxide (240 μM H_2O_2), in the presence of DTT (200 μM) as reducing agent, and according to the basic protocol IV reported in Kimberly et al, with minor modifications. (See supplementary materials for details). Enzymatic activity of Prx2 is expressed as $nmol_{H2O2}$ min^{-1} $nmol_{PRX2}^{-1}$ and it is the result of at least three different determinations. Sigma Plot software, version 10.0 (Jandel Scientific, San Rafael, CA, USA) was used for data analysis. The Student's t-test was applied to compare the difference in the enzymatic activity of the control vs phosphorylated form of Prx2.

Generation of Recombinant Prx2.

The cDNA sequence for human Prx2 was PCR-amplified using the sense primer 5′-CTCGAGATGGCCTCCGGTAA-3′, contains an XhoI site, and the antisense primer 5′-GGATCCCTAATTGTGTTTGGAGAAATA-3′, contains a BamHI restriction site. The resulting PCR products were subcloned in a TA cloning vector and ligated into a pET15b vector to produce WT Prx2. We have applied PCR based site-directed mutagenesis to change the codon of Tyr-193 of F. Prx2-Y193F was amplified by PCR using the WT Prx2 template, sense primer 5′-CTCGAGATGGCCTCCGGTAA-3′ and the antisense primer 5′-GGATCCCTAATTGTGTTTGGAGAAAAATTC-3′. The subsequent process is the same as the WT Prx2. We commissioned Bioneer (Korea Daejeon) to manufacture Prx2-Y115,193F mutant type and cloned the pET15b vector. Prx2-Y115F was amplified by PCR based site-directed mutagenesis using the Prx2-Y115,193F template and primer WT Prx2. The completed PCR product has a Tyr-115 of F. The subsequent process is the same as the WT.

In Vitro Activity of Recombinant Prx2.

Prx2 activity of recombinant enzyme was performed by measuring the decrease of hydrogen peroxide concentration, in the presence of DTT as reducing agent, and according to the basic protocol IV reported in Kimberly et al [11] with minor modifications.

Briefly: 2.5 μM Prx2 (in the buffer used for the phosphorylation experiments) was pre-treated with 250 μM DTT for 30 min at room temperature, for reduction and activation. Then, sample was diluted (*v/v*) with 0.1 M potassium phosphate buffer, pH 7.4, containing 100 mM ammonium sulfate and 1mM EDTA and transferred in a water bath at 37 °C. To allow for multiple turnover of Prx2, DTT was added to obtain 200 μM of final concentration and an aliquot of this sample (10 μL) was withdrawn and mix in the so named "Fox working reagent" (190 μL) (Fox working reagent: 1vol FOX A/100 vol FOX B; FOX A: 25mM ammonium ferrous sulfate in 2.5 M sulphuric acid; FOX B: 100 mM sorbitol and 125 μM xylenol orange). This sample was used as blank. Then, 240 μM H_2O_2 was added to start the enzymatic Prx2 reaction. The hydrogen peroxide concentration during this reaction was quantified at various time intervals (six time points in 10 min of total reaction time), by adding and mixing 10 μL of this assay solution to 190 μL of the FOX working reagent. After 30 min of incubation at room temperature, the absorbance at 560 nm of these samples were read. A standard curve with hydrogen peroxide was built, in the same experimental conditions and was used to calculate the concentration of H_2O_2 in the samples at the various time intervals. By plotting the hydrogen peroxide concentration (y-axis) *vs* time (x-axis), the rate of disappearance of H_2O_2, was calculated by linear regression analysis. A sample containing DTT, hydrogen peroxide and in absence of Prx2 was run under the same experimental condition to calculate the rate of the non-enzymatic decrease of H_2O_2 that was found to be negligible in our experimental conditions. Enzymatic activity of Prx2 is expressed as nmol H2O2 min^{-1} nmol $PRX2^{-1}$ and it is the result of at least three different determinations. Sigma Plot software, version 10.0 (Jandel Scientific, San Rafael, CA, USA) was used for data analysis. The Student's t-test was applied to compare the difference in the enzymatic activity of the control vs phosphorylated form of Prx2. The validity of this method, which also proved cost-effective as for the materials used and

adequate so as to assess the role of phosphorylation in altering Prx2 activity, was verified by performing experiments at fixed DTT concentration (200 μM) and varying hydrogen peroxide concentrations and confirmed by the results shown in Figure S3.

Mass Spectrometry.

Mass spectrometric analysis was performed using a Tofspec SE (Micromass, Manchester, UK) equipped with a delayed extraction unit. Peptide desorption was achieved using a laser wavelength of 337 nm, and mass spectra were obtained in the reflectron mode in the mass range 800–4000 Da. Peptide solutions were prepared with an equal volume of saturated alpha-cyano-4 hydroxycinnamic acid solution containing 40%acetonitrile-0.1% trifluoroacetic acid (*v/v*). External calibration was performed usingfragment ions from standard peptides, adrenocorticotropic hormone 18–39 and angiotensinI. Each mass spectrum was generated by accumulating data from 100–120 laser pulses.Database searches of peptide masses were performed using the search program "Mascot,Peptide Mass Fingerprint" (available at http://www.matrixscience.com). The followingsearch criteria were used: taxa Rodentia-Mus Musculus protein molecular mass rangefrom 10 to 300 kDa, trypsin digest, monoisotopic peptide masses, one missed cleavage by trypsin and a mass deviation of 100 ppm allowed in the NCBI database searches. Database searches of peptide masses were performed using the search program "Mascot,Peptide Mass Fingerprint" (available at http://www.matrixscience.com) as previously described.

Prx2 phosphorylation sites were analysed by a LTQ Orbitrap XL™ Hybrid Ion Trap-Orbitrap Mass Spectrometer (Thermo Fisher Scientific, Bremen, Germany). Briefly protein spots from SDS-PAGE gels were distained by repetitive washes with 100 mM NH4HCO3 (AMBIC) pH 8.0 and acetonitrile (ACN). Reduction and alkylation of cysteines was performed by 10mM dithiothreitol (DTT) (Sigma-Aldrich) in AMBIC 100 mM for 45 min at 56 °C followed by incubation with 55mM iodoacetamide (IAM) (Fluka) at room temperature for 30 min in the dark. Tryptic digestion (10 ng/μl) was carried out in 10 mM AMBIC pH 7.8 for 18 h at 37 °C. The resulting peptides mixture was dried and then dissolved in formic acid 0.1 % for MS analysis. Subdigestion with endoproteinase Asp-N was carried out in AMBIC 50mM pH 8.0 buffer at 37 °C for 18 h using a 1:50 *w/w* E/S ratio. The sample was incubated. Enrichment of phosphorylated peptides was performed using titanium dioxide coated magnetic beads (PHOS-TRAP™ , Perkin Elmer). The peptide mixture was incubated with the pre-equilibrated beads in Binding Buffer, the supernatant containing the unbound peptides was discarded and the retained phosphopeptides eluted in the Elution Buffer. Peptide mixtures were directly analysed by a LTQ Orbitrap XL™ Hybrid Ion Trap-Orbitrap Mass Spectrometer (Thermo Fisher Scientific, Bremen, Germany). Mass spectral data were used for protein identification with a licensed version of the Mascot Software (www.matrixscience.com). Identification of Prx2 phosphorylation sites following incubation with either Syk or Fyn was obtained by LC-MS/MS analyses of the corresponding tryptic and/or trypsin and AspN digests followed by manual inspection of the phosphopeptides MS/MS spectra.

References

1. Park, S. Y.; Matte, A.; Jung, Y.; Ryu, J.; Anand, W. B.; Han, E. Y.; Liu, M.; Carbone, C.; Melisi, D.; Nagasawa, T.; Locascio, J. J.; Lin, C. P.; Silberstein, L. E.; De Franceschi, L., Pathologic angiogenesis in the bone marrow of humanized sickle cell mice is reversed by blood transfusion. *Blood* **2020,** *135* (23), 2071-2084.
2. Matte, A.; Recchiuti, A.; Federti, E.; Koehl, B.; Mintz, T.; El Nemer, W.; Tharaux, P. L.; Brousse, V.; Andolfo, I.; Lamolinara, A.; Weinberg, O.; Siciliano, A.; Norris, P. C.; Riley, I. R.; Iolascon, A.; Serhan, C. N.; Brugnara, C.; De Franceschi, L., Resolution of sickle cell disease-associated inflammation and tissue damage with 17R-resolvin D1. *Blood* **2019,** *133* (3), 252-265.
3. Ferru, E.; Pantaleo, A.; Carta, F.; Mannu, F.; Khadjavi, A.; Gallo, V.; Ronzoni, L.; Graziadei, G.; Cappellini, M. D.; Turrini, F., Thalassemic erythrocytes release microparticles loaded with hemichromes by redox activation of p72Syk kinase. *Haematologica* **2014,** *99* (3), 570-8.
4. De Franceschi, L.; Tomelleri, C.; Matte, A.; Brunati, A. M.; Bovee-Geurts, P. H.; Bertoldi, M.; Lasonder, E.; Tibaldi, E.; Danek, A.; Walker, R. H.; Jung, H. H.; Bader, B.; Siciliano, A.; Ferru, E.; Mohandas, N.; Bosman, G. J., Erythrocyte membrane changes of chorea-acanthocytosis are the result of altered Lyn kinase activity. *Blood* **2011,** *118* (20), 5652-63.

5. Ura, B.; Monasta, L.; Arrigoni, G.; Licastro, D.; Di Lorenzo, G.; Romano, F.; Gaita, B.; Scrimin, F.; Ricci, G., Leiomyoma phosphoproteins involved in inhibition of oxidative stress and synthesis of reactive oxygen species. *Int J Mol Med* **2019,** *44* (6), 2329-2335.
6. Thingholm, T. E.; Larsen, M. R., Sequential Elution from IMAC (SIMAC): An Efficient Method for Enrichment and Separation of Mono- and Multi-phosphorylated Peptides. *Methods Mol Biol* **2016,** *1355*, 147-60.
7. de Franceschi, L.; Turrini, F.; Honczarenko, M.; Ayi, K.; Rivera, A.; Fleming, M. D.; Law, T.; Mannu, F.; Kuypers, F. A.; Bast, A.; van der Vijgh, W. J.; Brugnara, C., In vivo reduction of erythrocyte oxidant stress in a murine model of beta-thalassemia. *Haematologica* **2004,** *89* (11), 1287-98.
8. Matte, A.; Low, P. S.; Turrini, F.; Bertoldi, M.; Campanella, M. E.; Spano, D.; Pantaleo, A.; Siciliano, A.; De Franceschi, L., Peroxiredoxin-2 expression is increased in beta-thalassemic mouse red cells but is displaced from the membrane as a marker of oxidative stress. *Free Radic Biol Med* **2010,** *49* (3), 457-66.
9. Matte, A.; Bertoldi, M.; Mohandas, N.; An, X.; Bugatti, A.; Brunati, A. M.; Rusnati, M.; Tibaldi, E.; Siciliano, A.; Turrini, F.; Perrotta, S.; De Franceschi, L., Membrane association of peroxiredoxin-2 in red cells is mediated by the N-terminal cytoplasmic domain of band 3. *Free Radic Biol Med* **2013,** *55*, 27-35.
10. Franco, S. S.; De Falco, L.; Ghaffari, S.; Brugnara, C.; Sinclair, D. A.; Matte, A.; Iolascon, A.; Mohandas, N.; Bertoldi, M.; An, X.; Siciliano, A.; Rimmele, P.; Cappellini, M. D.; Michan, S.; Zoratti, E.; Anne, J.; De Franceschi, L., Resveratrol accelerates erythroid maturation by activation of FoxO3 and ameliorates anemia in beta-thalassemic mice. *Haematologica* **2014,** *99* (2), 267-75.
11. Nelson, K. J.; Parsonage, D., Measurement of peroxiredoxin activity. *Curr Protoc Toxicol* **2011,** *Chapter 7*, Unit7.10.
